# Long noncoding RNA AC003092.1 promotes temozolomide chemosensitivity through miR-195/TFPI-2 signaling modulation in glioblastoma

**DOI:** 10.1038/s41419-018-1183-8

**Published:** 2018-11-15

**Authors:** Ningbo Xu, Boyang Liu, Changlin Lian, Desislava Met Doycheva, Zhao Fu, Yanting Liu, Jian Zhou, Zhenyan He, Zhao Yang, Qiongzhen Huang, Huijun Zeng, Hongbo Guo

**Affiliations:** 1 Department of Neurosurgery, The National Key Clinical Specialty, The Engineering Technology Research Center of Education Ministry of China, Guangdong Provincial Key Laboratory on Brain Function Repair and Regeneration, Zhujiang Hospital, Southern Medical University, Guangzhou, 510282 China; 2 Department of Physiology and Pharmacology, Basic Sciences, School of Medicine, Loma Linda University, Loma Linda, CA 92354 USA

## Abstract

Temozolomide (TMZ) and radiation therapy combination for glioblastoma (GB) patients has been considered as the most effective therapy after surgical procedure. However, the overall clinical prognosis remains unsatisfactory due to intrinsic or developing resistance to TMZ. Recently, increasing evidence suggested that long noncoding RNAs (lncRNAs) play a critical role in various biological processes of tumors, and have been implicated in resistance to various drugs. However, the role of lncRNAs in TMZ resistance is poorly understood. Here, we found that the expression of lncRNA AC003092.1 was markedly decreased in TMZ resistance (TR) of GB cells (U87TR and U251TR) compared with their parental cells (U87 and U251). In patients with glioma, low levels of lncRNA AC003092.1 were correlated with increased TMZ resistance, higher risk of relapse, and poor prognosis. Overexpression of lncRNA AC003092.1 enhances TMZ sensitivity, facilitates cell apoptosis, and inhibits cell proliferation in TMZ-resistant GB cells. In addition, we identified that lncRNA AC003092.1 regulates TMZ chemosensitivity through TFPI-2-mediated cell apoptosis in vitro and in vivo. Mechanistically, further investigation revealed that lncRNA AC003092.1 regulates TFPI-2 expression through miR-195 in GB. Taken together, these data suggest that lncRNA AC003092.1 could inhibit the function of miR-195 by acting as an endogenous CeRNA, leading to increased expression of TFPI-2; this promotes TMZ-induced apoptosis, thereby making GB cells more sensitive to TMZ. Our findings indicate that overexpression of lncRNA AC003092.1 may be a potential therapy to overcome TMZ resistance in GB patients.

## Introduction

Glioblastoma (GB) is one of the most aggressive primary brain tumors in adults with widespread invasion and resistance to traditional treatments^1,2^. Currently, temozolomide (TMZ)-based chemotherapy after surgical excision is one of the most frequently used therapeutic strategies for GB patients^3,4^. Unfortunately, a large proportion of patients developing resistance to TMZ becomes the major barrier to the efficacy of GB treatment^5–7^. It has been well documented that the relative expression of DNA repair protein, O6-methylguanine-DNA methyltransferase (MGMT), determines the response to TMZ^8–10^. MGMT removes cytotoxic lesions generated by TMZ, and its promoter methylation is correlated with improved overall survival and reduced progression in patients treated with TMZ^8–11^. However, only half of the patients with GB having MGMT promoter methylation respond to TMZ, indicating that MGMT is not the only factor contributing to TMZ resistance. Therefore, elucidation of molecular mechanisms underlying TMZ resistance could provide potential novel targets for GB treatments.

LncRNA represents a novel class of RNAs which were greater than 200 nucleotides in length without functional protein-coding ability^12–14^. Recently, several lines of evidence point to the functional role of dysregulated lncRNA in the cancer formation and progression, as well as the resistance to chemotherapy^15,16^. The lncRNA colorectal neoplasia differentially expressed (CRNDE) and cancer susceptibility candidate 2 (CASC 2) inhibits proliferation, migration, and invasion in glioma cells by increasing the expression of mTOR or decreasing the expression of miR-21^17^. Additionally, lncRNA H9 and RP11-838N2.4 have been reported to enhance cytotoxic effects of temozolomide in GB cell lines^18,19^. Thus, genomic characterization of lncRNA alterations may provide an alternative therapeutic strategy for TMZ-resistant GB. Previously, our microarray analysis showed 2,692 lncRNAs and 2,933 mRNAs exhibiting a change of more than 2.0-fold in TMZ-resistant U87 (U87TR) cells^20^. Of note, lncRNA AC003092.1 and its nearby gene, itssue factor pathway inhibitor-2 (TFPI-2), showed a remarkable downregulation in U87TR cells when compared with its parental U87 cells (43.99 folds and 607.05 folds, respectively)^20^. However, far less is known about the role of lncRNA AC003092.1-mediated regulation of TMZ resistance in GB as well as the underlying mechanism.

It is known that lncRNAs simultaneously regulate the expression of one or several spatially proximal genes^21,22^. Thus, the significantly low expression of TFPI-2 in U87TR may be the result of downregulation of lncRNA AC003092.1. TFPI-2 is a serine protease inhibitor which is abundant in the extracellular matrix. Low expression of TFPI-2 correlates with the poor prognosis of human gliomas^23^. Overexpression of TFPI-2 could inhibit cell migration^24^, proliferation^25^, and promote cell apoptosis^26^ in glioma cells. Moreover, TFPI-2 inhibits the function of P-glycoprotein efflux pump, resulting in reduced TMZ efflux in GB cells^27^. However, whether TFPI-2 is the potential target of lncRNA AC003092.1 in TMZ-resistant GB remains unclear.

Currently, the competing endogenous RNA (ceRNA) hypothesis has been proposed to describe the cross talk of lncRNAs with their responsible coding gene^28,29^. Accumulating evidence suggests that lncRNAs act as a natural miRNA sponge to de-repress its target gene by competitively binding miRNA^30^. It is confirmed that miR-195, a putative target of lncRNA AC003092.1 and TFPI-2 predicted by Starbase2.0 based on a base-pairing principle, is involved in the regulation of TMZ resistance of GB cells. Additionally, knockdown of miR-195 with TMZ treatment strongly enhances its toxic effect on glioblastoma cells, indicating that miR-195 plays a vital role in TMZ resistance.

Collectively, we hypothesized that lncRNA AC003092.1 participated in the enhancement of TMZ sensitivity by competitively sponging and then inhibiting miR-195 to augment TFPI-2 expression in GB cells. First, we evaluated the expression of lncRNA AC003092.1 in glioma tissues, GB cell lines, and estimated its clinical relevance. Next, we explored the role of lncRNA AC003092.1 and its genomic neighboring gene TFPI-2 on GB cell proliferation and apoptosis. Finally, we tested whether miR-195 was involved in lncRNA AC003092.1-mediated regulation of TFPI-2. Thus, our research will shed light on the characterization of lncRNA AC003092.1 as an underlying therapeutic target to reverse the TMZ resistance in patients with glioma.

## Results

### LncRNA AC003092.1 downregulation correlates to TMZ resistance and poor prognosis in glioma

**Fig. 1:**
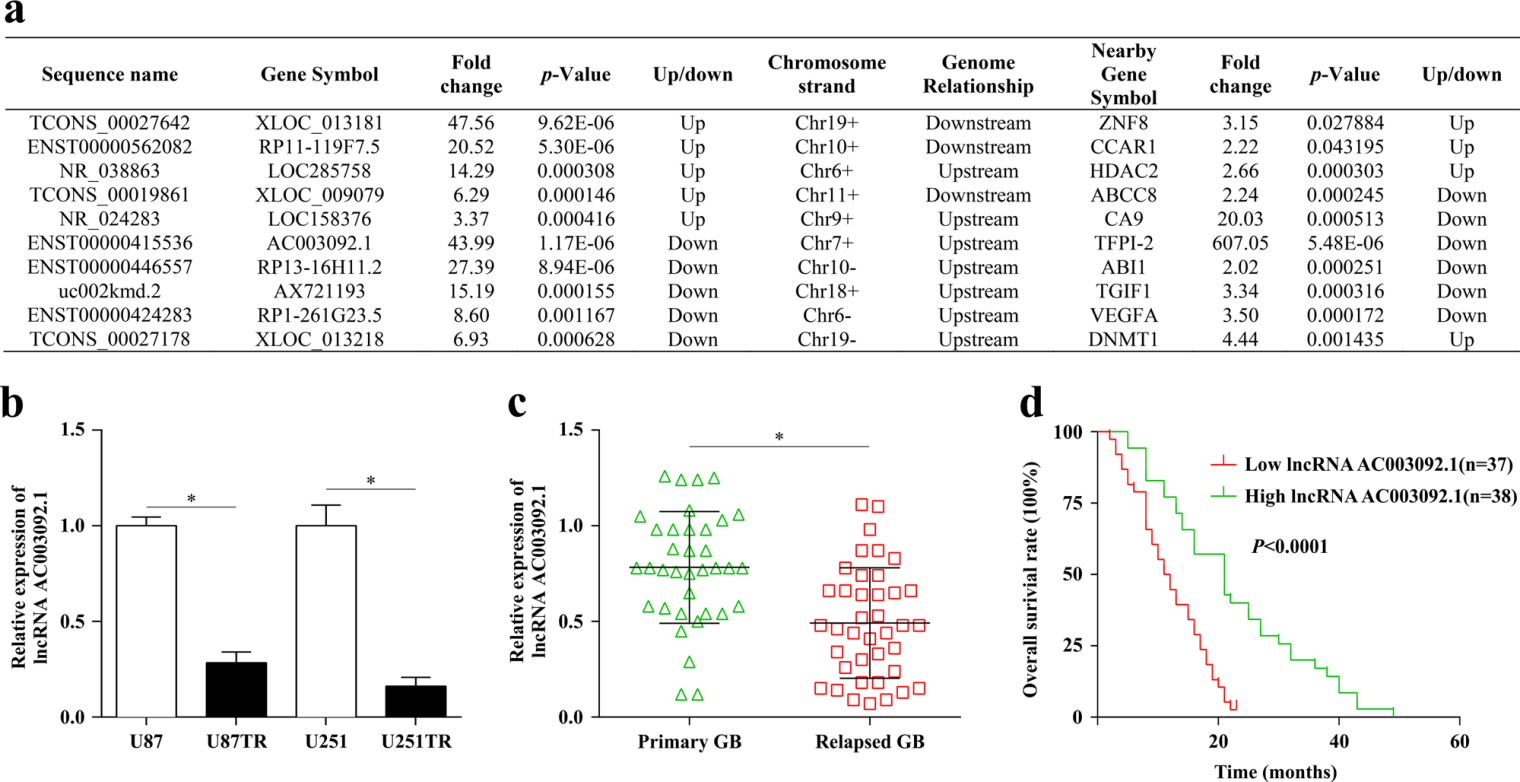
Expression of lncRNA AC003092.1 in GB cell lines and glioma tissues and its clinical significance. **a** Top five significantly up- and downregulated lncRNAs and mRNA expression of their nearby genes (distance < 300 kb) in U87TR cells. **b** lncRNA AC003092.1 expression level in TMZ-resistant and its parental GB cells, ^*^P < 0.05 compared with U87 or U251 cells. **c** lncRNA AC003092.1 expression in primary and relapsed GB tissues was examined by qRT-PCR and normalized against U6 expression, ^*^P < 0.05 compared with primary GB tissues. **d** Kaplan–Meier overall survival curves according to high and low lncRNA AC003092.1 expression, *P* < 0.0001. lncRNA AC003092.1 expression level was assessed by qRT-PCR, and the median value for all 75 GB cases was chosen as the cutoff point with which the cases were separated into high (*n* = 37) and low (*n* = 38) lncRNA AC003092.1 expression group. The patients were classified into two groups according to lncRNA AC003092.1 expression; Kaplan–Meier overall survival curves according to lncRNA AC003092.1 expression level, *P* < 0.0001. Data are presented as the mean ± SD of three independent experiments

Using microarray methods, we previously explored the global expression profiles of lncRNAs and mRNAs in the parental U87 and the U87TR (TMZ-resistant) cells^19,20^. Among the differentially expressed lncRNAs, we found that the lncRNA AC003092.1 with 639-nucleotide (nt) length, known as ENST00000415536 (chr7: 93652144–93694035), was mostly downregulated with 43.99-fold, while its nearby gene TFPI-2 was downregulated with 607.05-fold change within U87TR cells, as compared with its parental U87 cells (Fig. 1a). To verify this, the qRT-PCR analysis was applied, and the results showed that lncRNA AC003092.1 expression was significantly downregulated in both U87TR and U251TR cells, as compared to their parental cells in vitro (Fig. 1b). For further validation in vivo, 108 surgical resection specimens from glioma patients who are receiving neoadjuvant chemotherapy were analyzed for the lncRNA AC003092.1 expression and the clinicopathological characteristics through qRT-PCR assay. As shown in Table 1, no significant association was observed between the lncRNA AC003092.1 expression and patient’s sex or age, while the lncRNA AC003092.1 expression was negatively correlated with the tumor grading (WHO I/II versus WHO III/IV) (*P* < 0.001, Mann–Whitney test). Furthermore, we also found that the relapsed glioma patients with 6 months of TMZ chemotherapy showed a relatively lower lncRNA AC003092.1 expression than that of primary glioma patients (Fig. 1c). In addition, glioma patients with higher lncRNA AC003092.1 expression obtained a longer median survival time and better prognosis when compared to the glioma patients with lower lncRNA AC003092.1 expression (Fig. 1d). These data suggested that lncRNA AC003092.1 downregulation correlates to TMZ resistance and the poor prognosis in glioma.

**Table 1:** Association between lncRNA AC003092.1 expression and the clinicopathological characteristics of 108 patients with glioma

Characteristics	No. of patients (*n* = 108)	Low expression (*n* = 49)	High expression (*n* = 59)	*P*
*Gender*				
Male	59 (54.6%)	25 (42.4%)	34 (57.6%)	0.90
Female	49 (45.4%)	24 (49.0%)	25 (51.0%)	
*Age, years*				
< 50	25 (23.1%)	8 (32.0%)	17 (68.0%)	0.24
≥50	83 (76.9%)	41 (49.4%)	42 (50.6%)	
*WHO grade*				
I/II	28 (25.9%)	9 (32.1%)	19 (67.9%)	0.00*
III/IV	80 (74.1%)	40 (50.0%)	40 (50.0%)	

*WHO*World Health Organization

*Statistical significance (*P* < 0.05)

### LncRNA AC003092.1 overexpression enhances TMZ sensitivity, facilitates cell apoptosis, and inhibits cell proliferation in TMZ-resistant GB cells

**Fig. 2:**
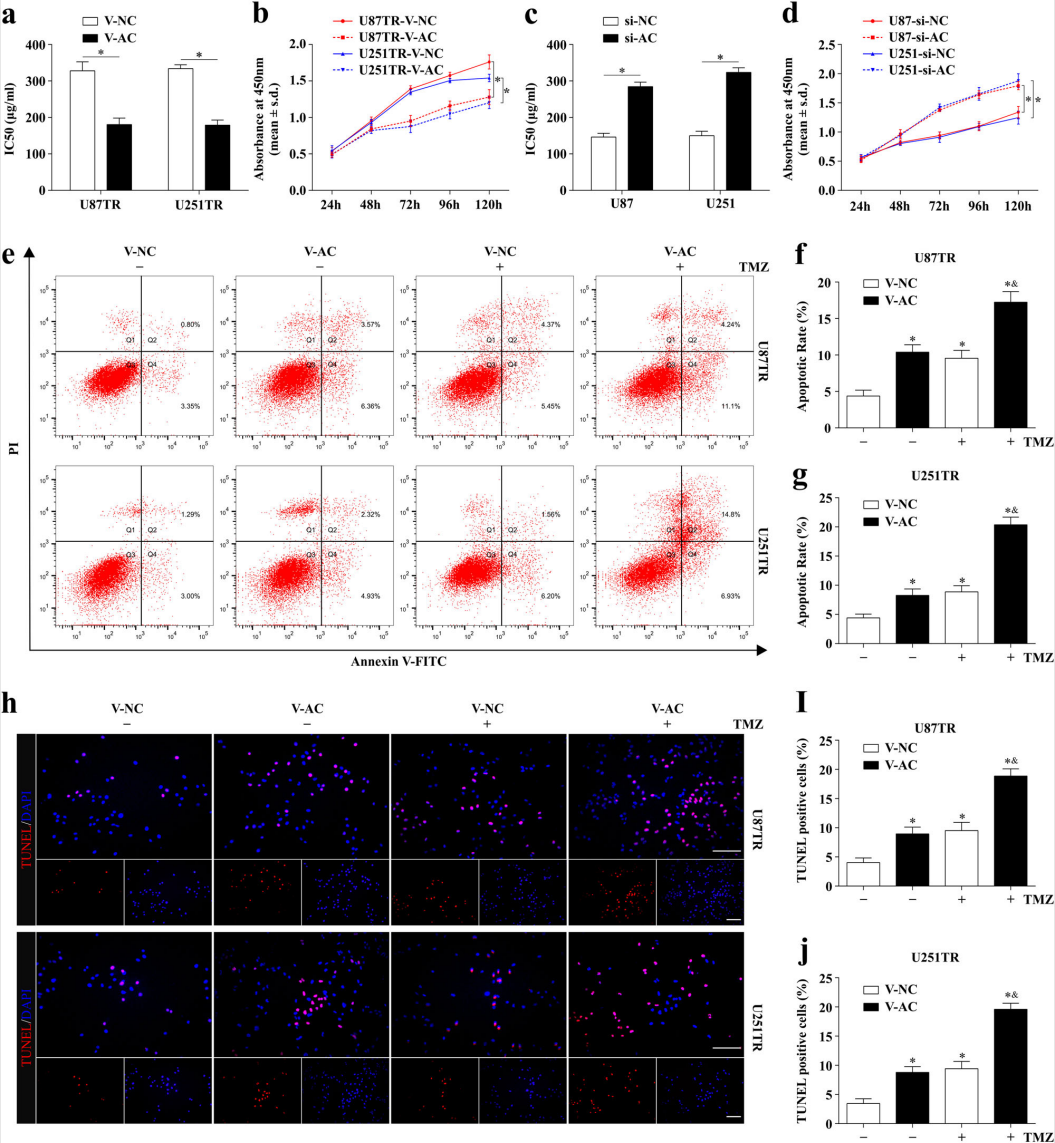
Effects of lncRNA AC003092.1 on TMZ sensitivity, cell apoptosis, and proliferation in GB cells. **a** The TMZ sensitivity of U87TR and U251TR cells after V-NC or V-AC transfection measured by CCK-8 assay. ^*^P < 0.05 compared with V-NC group cells. **b** Cell viability of U87TR and U251TR V-NC or V-AC cells treated with 50 μg/ml TMZ for 24, 48, 72, 96, and 120 h. ^*^P < 0.05 compared with V-NC group cells. **c** The TMZ sensitivity of U87 and U251 cells after treatment of si-NC or si-AC. ^*^P < 0.05 compared with si-NC group cells. **d** Cell viability of U87 and U251 si-NC or si-AC cells treated with 50 μg/ml TMZ for 24, 48, 72, 96, and 120 h. ^*^P < 0.05 compared with si-NC group cells. The rate of apoptosis of U87TR and U251TR V-NC or V-AC cells after treatment with or without 50 μg/ml TMZ for 48 h determined by FACS-based Annexin-V/FITC double staining (**e**–**g**) and TUNEL (**h**–**j**). ^*^P < 0.05 compared with V-NC cells without TMZ treatment, ^&^*P* < 0.05 compared with V-NC group cells with TMZ treatment. Scale bar = 50 μm. Data are presented as mean ± SD of three independent experiments

Since lncRNA AC003092.1 downregulation correlates to TMZ resistance, we asked whether lncRNA AC003092.1 overexpression could enhance TMZ chemosensitivity in glioma cells. To this end, U87TR and U251TR cells with stable lncRNA AC003092.1 expression (U87TR-V-AC, U251TR-V-AC) were constructed after V-AC (virus-lncRNA AC003092.1) or V-NC (virus-negative control) transfection (Supplementary Figure 1a, b), while for lncRNA AC003092.1 knockdown, U87 and U251 cells were transfected with si-AC or si-NC, respectively (Supplementary Figure 1c–d). Through CCK-8 assay, we found that lncRNA AC003092.1 overexpression significantly decreased the cell viability in U87TR and U251TR cells with relatively lower IC_50_ values, as compared to the V-NC groups treated with 50 μg/ml TMZ for 48 h (Fig. 2a, b). In contrast, increased cell viability and a relatively higher IC_50_ value were observed in lncRNA AC003092.1-knockdown U87 and U251 cells when compared to the si-NC groups (Fig. 2c, d). To further evaluate the lncRNA AC003092.1 expression on cell survival, cell apoptosis analysis by flow cytometry was applied in TR cells treated with or without 50 μg/ml TMZ for 48 h. The results showed only (4.37 ± 0.80)% of U87TR and (4.40 ± 0.66)% of U251TR V-NC-treated cells that underwent either apoptosis or death compared with (10.40 ± 1.01)% U87TR and (8.27 ± 1.10)% U251TR V-AC groups without TMZ treatment (Fig. 2e–g). Furthermore, the rate of apoptosis was significantly increased in V-AC groups when compared with V-NC groups with TMZ treatment. Similarly, the TUNEL assay showed that the percentage of apoptotic cells was also higher in TMZ-treated V-AC groups than that of V-NC groups in U87TR and U251TR cells (Fig. 2h–j). Additionally, the effect of lncRNA AC003092.1 expression on cell proliferation was further assessed by EdU assay. The percentage of EdU-positive cells was significantly decreased in V-AC groups when compared with that of V-NC groups, which were more evident after TMZ treatment (Supplementary Figure 1e–g). Together, these data indicated that lncRNA AC003092.1 overexpression enhances TMZ sensitivity, facilitates cell apoptosis, and inhibits cell proliferation in TMZ-resistant GB cells.

### TFPI-2 is the potential target of lncRNA AC003092.1

**Fig. 3:**
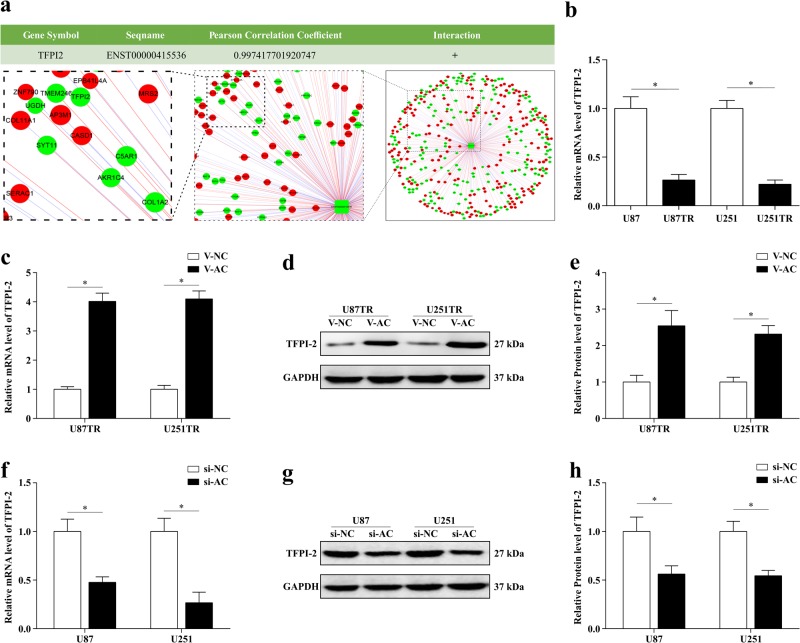
TFPI-2 is the potential target of lncRNA AC003092.1. **a** Co-expression analysis between protein-coding RNAs and lncRNA AC003092.1(ENST00000415536). Expression of TFPI-2 was highly positively correlated to lncRNA AC003092.1 expression. **b** mRNA level of TFPI-2 in U87, U87TR, U251, and U251TR cells measured by qRT-PCR. ^*^P < 0.05 compared with U87 or U251 cells. TFPI-2 mRNA (**c**) and protein expression (**d, e**) in U87TR and U251TR after V-NC or V-AC transfection. ^*^P < 0.05 compared with V-NC group cells. Expression of TFPI-2 mRNA (**f**) and protein (**g, h**) in U87 and U251 treated with si-NC or si-AC determined by qRT-PCR. ^*^P < 0.05 compared with si-NC group cells. Data are presented as mean ± SD of three independent experiments

To further explore the molecular mechanisms of lncRNA AC003092.1 in regulating glioma TMZ chemosensitivity, the co-expression analysis between protein-coding RNAs and lncRNA AC003092.1 was conducted. The results showed that TFPI-2, a prognostic marker in GB^23^, was highly correlated to the ENST00000415536 (lncRNA AC003092.1) expression (r = 0.997, Fig. 3a). Meanwhile, the UCSC Genome Browser database (https://genome.ucsc.edu/) also revealed that TFPI-2 located approximately 32 kb away from the lncRNA AC003092.1. In addition, the results of mRNA microarray showed that TFPI-2 was markedly downregulated (607.05-fold, Fig. 1a) in U87TR cells when compared with the parental U87 cells in which it was further identified by qRT-PCR (Fig. 3b)^20^. Given the similar co-expression profile in TMZ-resistant glioma cells and the close location relationship of lncRNA AC003092.1 and TFPI-2, we hypothesized whether lncRNA AC003092.1 regulated TMZ chemoresistance in glioma cells by targeting TFPI-2 expression. To this end, TPFI-2 expression was assessed in lncRNA AC003092.1 overexpression or knockdown in TMZ-resistant glioma cells through qRT-PCR and western blot, respectively. The results showed that lncRNA AC003092.1 overexpression significantly increased the TFPI-2 mRNA and protein expression levels in U87TR and U251TR cells (Fig. 3c–e), while TFPI-2 levels were downregulated in lncRNA AC003092.1-knockdown U87 and U251 cells (Fig. 3f–h). Therefore, these results validated that lncRNA AC003092.1 could regulate TFPI-2 expression in TMZ-resistant GB cells and TFPI-2 may act as a potential target of lncRNA AC003092.1 in regulating GB TMZ chemosensitivity.

### LncRNA AC003092.1 regulates TMZ chemosensitivity through TFPI-2- mediated cell apoptosis

**Fig. 4:**
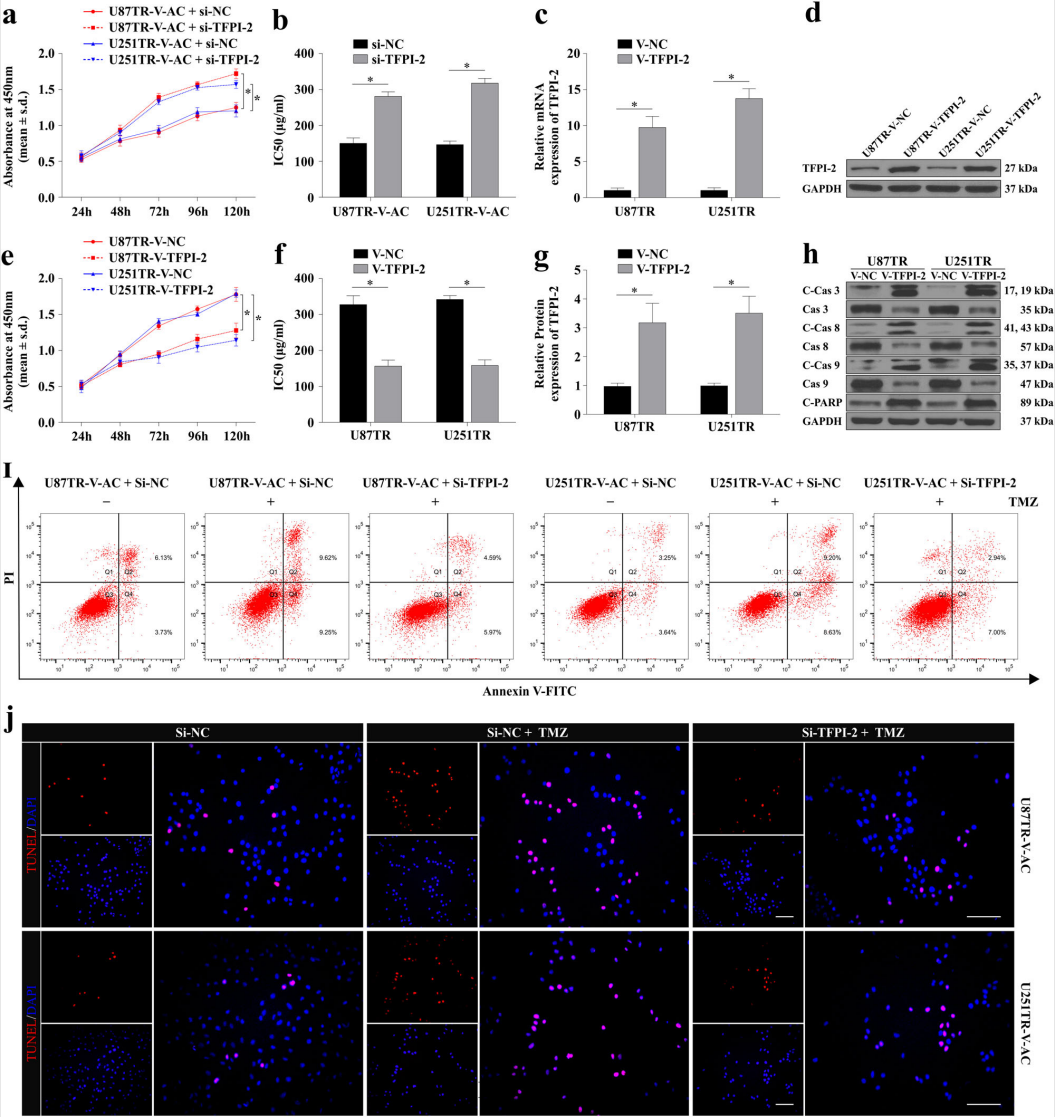
lncRNA AC003092.1 enhances TMZ sensitivity through TFPI-2- mediated cell apoptosis. **a** Cell viability of U87TR-V-AC and U251TR-V-AC cells after si-NC or si-TFPI-2 treatment measured by CCK-8 assay. ^*^P < 0.05 compared with U87TR-V-AC + si-NC cells or U251TR-V-AC + si-NC cells. **b** The TMZ sensitivity of U87TR-V-AC and U251TR-V-AC cells after intervention of si-NC or si-TFPI-2 determined by CCK-8 assay. ^*^P < 0.05 compared with si-NC-treated cells. The mRNA (**c**) and protein (**d, g**) level of TFPI-2 in U87TR and U251TR after transfection of V-NC or V-TFPI-2. ^*^P < 0.05 compared with V-NC-treated cells. **e** Cell viability and (**f**) the TMZ sensitivity of U87TR and U251TR cells after V-NC or V-TFPI-2 transfection. ^*^P < 0.05 compared with V-NC-treated cells. **h** The protein level of proapoptotic proteins (cleaved caspase 3, cleaved caspase 8, cleaved caspase 9, and cleaved PARP) after V-NC or V-TFPI-2 treatment in U87TR and U251TR cells. ^*^P < 0.05 compared with V-NC-treated cells. The ratio of apoptosis of U87TR-V-AC and U251TR-V-AC cells after si-NC or si-TFPI-2 transfection with or without 50 μg/ml TMZ treatment for 48 h determined by flow cytometry (**i**) and TUNEL (**j**). ^*^P < 0.05 compared with si-NC-treated cells. ^&^*P* < 0.05 compared with si-NC +TMZ-treated cells. Scale bar = 50 μm. Data are presented as mean ± SD of three independent experiments

Recent studies have demonstrated that the TFPI-2, a Kunitz-type proteinase inhibitor, could inhibit cell migration, proliferation, and promote cell apoptosis in glioma cells^24–26^. First, to test whether TFPI-2 was involved in TMZ chemosensitivity regulation by lncRNA AC003092.1, TFPI-2 siRNA was co-transfected into the U87TR-V-AC and U251TR-V-AC cells, respectively. Through the CCK-8 assay, we found that TFPI-2 interference significantly decreased cell viability and reduced the lncRNA AC003092.1-mediated TMZ sensitivity in V-AC co-transfected TMZ-resistant cells compared with that of the si-NC group (Fig. 4a, b). Then we sought to investigate the role of TFPI-2 in TMZ sensitivity regulation. After stable V-TFPI-2 transfection (Fig. 4c–g), we found that TFPI-2 upregulation significantly decreased the cell viability of U87TR and U251TR cells (Fig. 4e, f). Moreover, TFPI-2 overexpression dramatically increased the proapoptotic protein cleaved caspase-3, cleaved caspase-7, cleaved caspase-9, and cleaved PARP expression, as compared with the group of V-NC in U87TR and U251TR cells (Fig. 4h, Supplementary Figure 2a). In addition, the flow cytometric analysis and TUNEL assays showed that TFPI-2 inhibition decreased cell apoptosis in V-AC co-transfected TMZ-resistant cells (Fig. 4i, j and Supplementary Figure 2b–c). Together, these above data confirmed that lncRNA AC003092.1 regulates TMZ chemosensitivity through TFPI-2-mediated cell apoptosis in glioma cells.

### LncRNA AC003092.1 functions as ceRNA and negatively modulates miR-195 expression

**Fig. 5:**
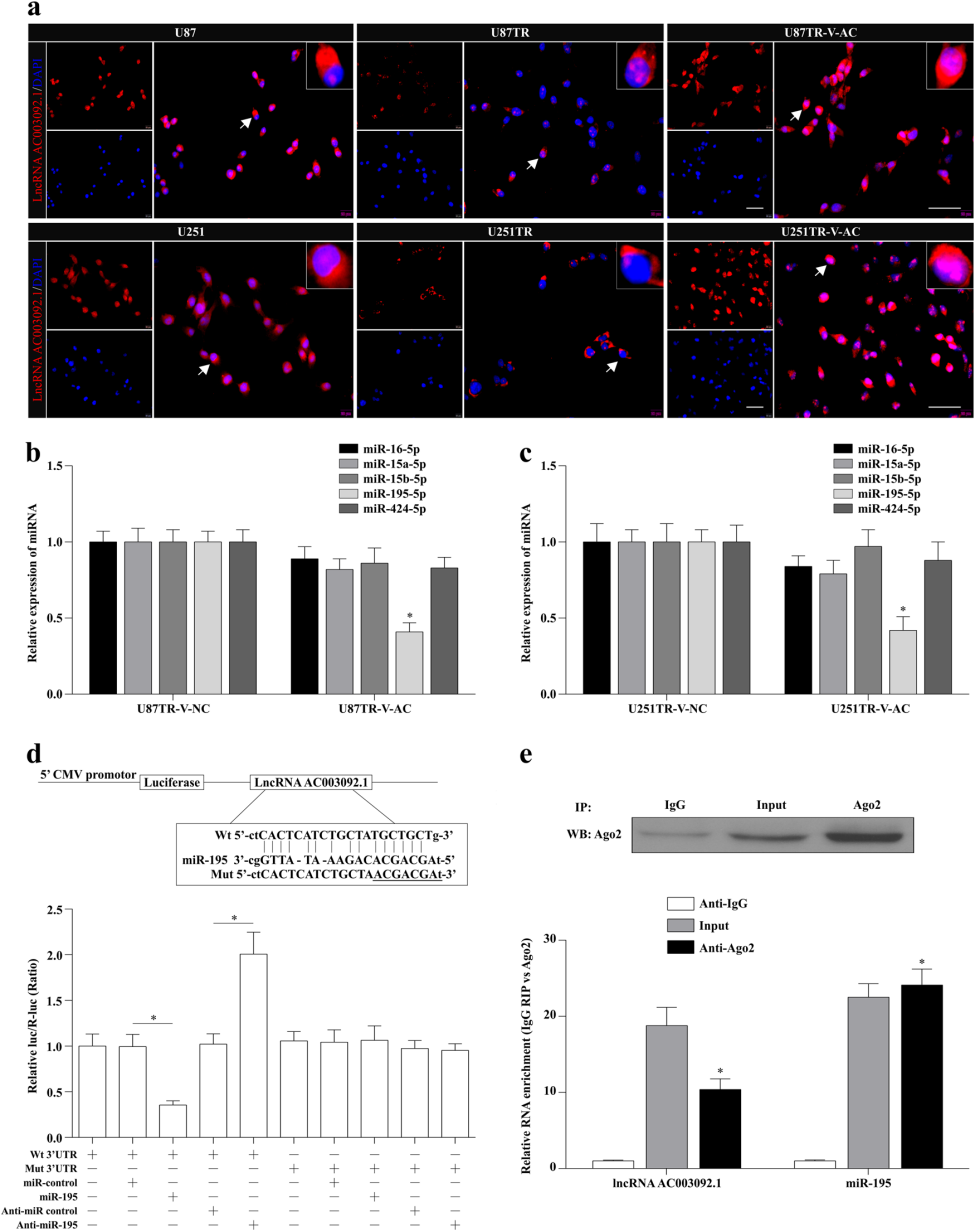
lncRNA AC003092.1 functions as ceRNA and negatively modulates miR-195 expression. **a** Cellular localization of lncRNA AC003092.1 in U87, U87TR, U87TR-V-AC, U251, U251TR, and U251TR-V-AC cells by RNA-FISH assay. Scale bar = 50 μm. **b**, **c** Expression of miRNAs in U87TR and U251TR after V-NC or V-AC transfection measured by qRT-PCR. ^*^P < 0.05 compared with U87TR-V-NC or U251TR-V-NC cells. **d** The luciferase activity of pMIR-lncRNA AC003092.1-Wt and pMIR-lncRNA AC003092.1-Mut after miR-195 or anti-miR-195 transfection in U87TR cells by dual-luciferase reporter assay. ^*^P < 0.05. **e** The expression of lncRNA AC003092.1 and miR-195 in the complexes using anti-Ago2 antibody by RNA-binding protein immunoprecipitation assay. ^*^P < 0.05 compared with anti-IgG group. Data are presented as mean ± SD of three independent experiments

Accumulating evidence has demonstrated that lncRNAs are able to function as master regulators for gene expression through various mechanisms, such as regulation of transcription, post transcription, translation, protein modification, etc.^31–34^. Since the cellular localization and function of lncRNA AC003092.1 were not that clear at present, the specific lncRNA AC003092.1 probes were designed and applied in RNA-FISH assay. The results showed that lncRNA AC003092.1 was mainly located in the cytoplasm with a relatively high expression in U87 and U251 cells, as compared to its counterpart TR cells (Fig. 5a). Based on these data, we theorized that lncRNA AC003092.1 could function as a ceRNA to bind with miRNAs, leading to the change of downstream TFPI-2 expression^35^. To examine this notion, the Starbase 2.0 database (http://starbase.sysu.edu.cn/) was applied to predict for the potential miRNAs with the common binding site of both lncRNA AC003092.1 and TFPI-2. Bioinformatic prediction showed that the miR-16-5p, miR-15a-5p, miR-15b-5p, miR-195-5p, and miR-424-5p had putative binding sites with lncRNA AC003092.1 and TFPI-2 (Supplementary Table S1).

To investigate whether these potential miRNAs expression was correlated to the lncRNA AC003092.1, qRT-PCR assay was conducted, and the results showed that only the miR-195 expression was significantly downregulated after lncRNA AC003092.1 overexpression, as compared with the U87TR-V-NC and U251TR-V-NC cells (Fig. 5b, c). Since the lncRNA AC003092.1 contained one conserved binding site for miR-195, we then sub-cloned this conserved binding site with wild type (AC-Wt) or mutant type (AC-Mut) into the pMIR luciferase reporter, and co-transfected with miR-195 or anti-miR-195 in U87TR cells, respectively (Fig. 5d). The results showed that the luciferase activity of pMIR-lncRNA AC003092.1-Wt was significantly decreased after miR-195 transfection, while there was a relatively increased activity in pMIR-lncRNA AC003092.1-Wt after anti-miR-195 transfection in U87TR cells (Fig. 5d). In addition, the relative luciferase activity of pMIR-lncRNA AC003092.1-Mut was not affected after miR-195 or anti-miR-195 transfection (Fig. 5d). These suggest that lncRNA AC003092.1 is targeted by miR-195.

Since the miRNAs are present in the form of miRNA ribonucleoprotein complexes that contain the key component of the RNA-induced silencing complex (RISC) Ago2^36,37^, we next sought to determine the relationship between lncRNA AC003092.1 and the miR-195 through the RNA immunoprecipitation (RIP) with an anti-Ago2 antibody. As shown in Fig. 5e, the Ago2 protein was sufficiently immunoprecipitated from the cell extracts, and both the lncRNA AC003092.1 and miR-195 were significantly enriched by 10.4 ± 1.4 and 24.1 ± 2.1 folds in the anti-Ago2 group as compared to the anti-IgG group (Fig. 5e). Together, these results indicated that the lncRNA AC003092.1 acted as a miR-195 sponge in TFPI-2-mediated TMZ chemosensitivity regulation.

### lncRNA AC003092.1 regulates TFPI-2 expression through miR-195 in GB

**Fig. 6:**
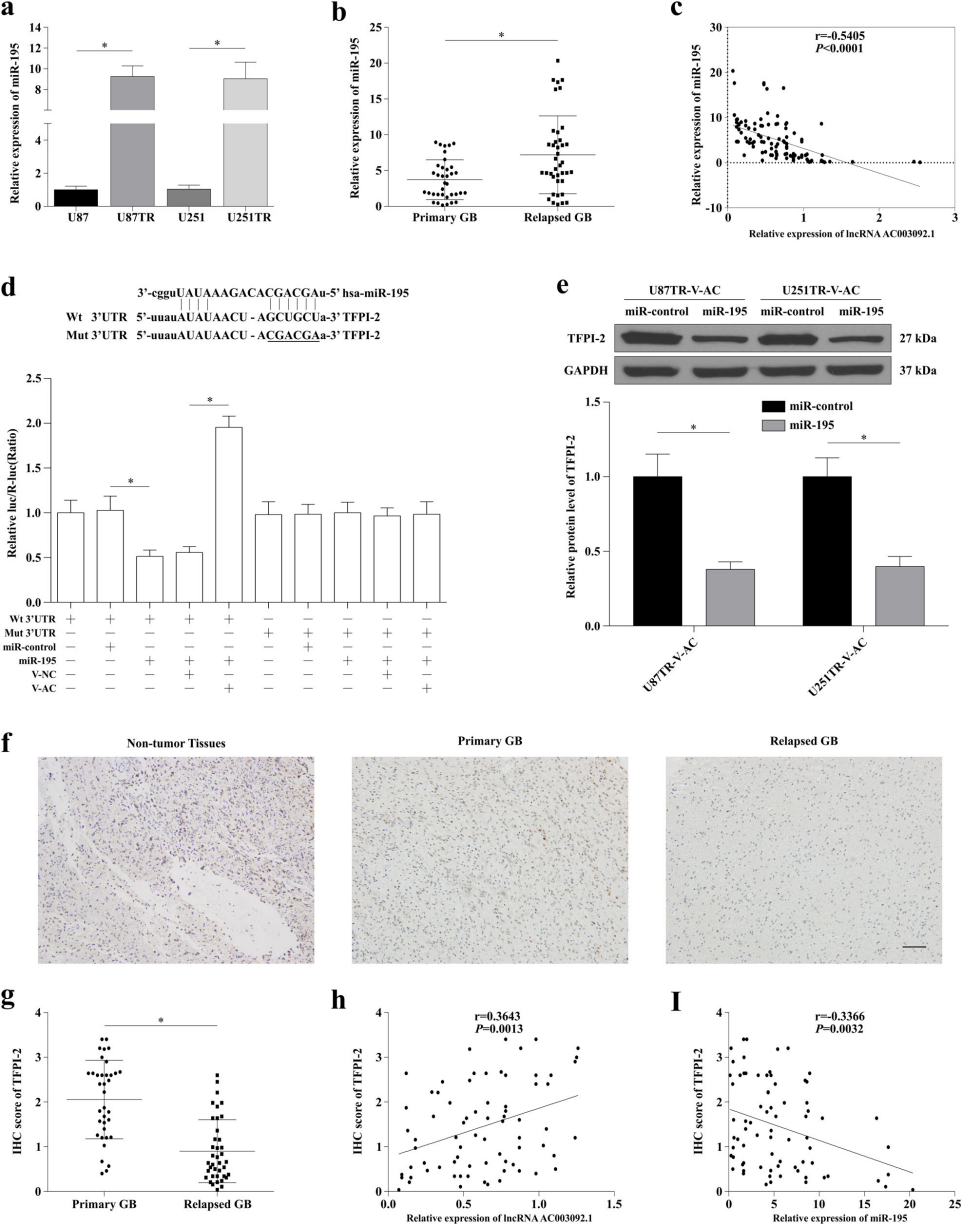
lncRNA AC003092.1 regulates GB TMZ chemosensitivity through miR-195/TFPI-2. **a** Expression of miR-195 in U87, U87TR, U251, and U251TR cells measured by qRT-PCR. ^*^P < 0.05 compared with U87 or U251 cells. **b** Expression of miR-195 in primary and relapsed GB tissues by qRT-PCR. ^*^P < 0.05 compared with primary GB tissues. **c** The relationship of lncRNA AC003092.1 and miR-195 expression in GB tissues through qRT-PCR analysis. **d** Dual-luciferase assay was performed in U87TR cells transfected with luciferase construct alone or co-transfected with miR-195 and V-AC. Firefly luciferase containing a wild or mutant target site of TFPI-2 was constructed and transfected as indicated. Firefly luciferase activity was normalized to Renilla luciferase activity for each sample. ^*^P < 0.05. **e** Expression of TFPI-2 protein level after miR-control or miR-195 transfection in U87TR-V-AC or U251TR-V-AC cells. ^*^P < 0.05 compared with miR-control treated cells. **f** Expression of TFPI-2 in non-tumor and GB tissues by immunohistochemistry (IHC) assay. Scale bar = 100 μm. **g** IHC scores of TFPI-2 in primary and relapsed GB tissues. ^*^P < 0.05 compared with primary GB tissues. **h, i** The correlation analysis of TFPI-2 IHC scores and lncRNA AC003092.1 (**h**) or miR-195 (**i**) expression and in GB tissues. Data are presented as mean ± SD of three independent experiments

We then investigated the levels of miR-195 expression in GB tissues and cell lines. As shown in Fig. 6a, the expression levels of miR-195 were drastically higher in both U87TR and U251TR cell lines, which showed an opposite trend to lncRNA AC003092.1 expression level. Moreover, miR-195 expression was higher in relapsed GB tissues compared to primary GB tissues (Fig. 6b) and an inverse correlation was noted between miR-195 and lncRNA AC003092.1 (Fig. 6c).

To confirm the effect of miR-195 on TFPI-2, human TFPI-2 3′-UTR fragments containing putative binding sites for miR-195 reporter vector were adopted for dual-luciferase reporter assay. The results indicated that the relative TFPI-2-3′-UTR luciferase activity was significantly reduced in U87TR cells transfected with miR-195 compared with miR-control (Fig. 6d). However, co-transfection with miR-195 and V-AC or V-NC, increased luciferase activity, which was observed in miR-195+TFPI-2-3′-UTR-Wt+V-AC when compared with miR-195+TFPI-2-3′-UTR-Wt+V-NC (Fig. 6d). In addition, upregulation of miR-195 with miR-195 mimics efficiently reversed the increased TFPI-2 protein level induced by V-AC in U87TR and U251TR cells (Fig. 6e).

Finally, the protein expression of TFPI-2 was measured in primary GB, relapsed GB, and non-tumor primary GB tissues (Fig. 6f). The expression of TFPI-2 was lower in relapsed GB tissues compared to primary GB tissues (Fig. 6g). Meanwhile, TFPI-2 expression was positively correlated with lncRNA AC003092.1 expression (Fig. 6h), and negatively associated with miR-195 expression in GB tissues (Fig. 6i).

### LncRNA AC003092.1 overexpression enhances TMZ sensitivity in vivo

**Fig. 7:**
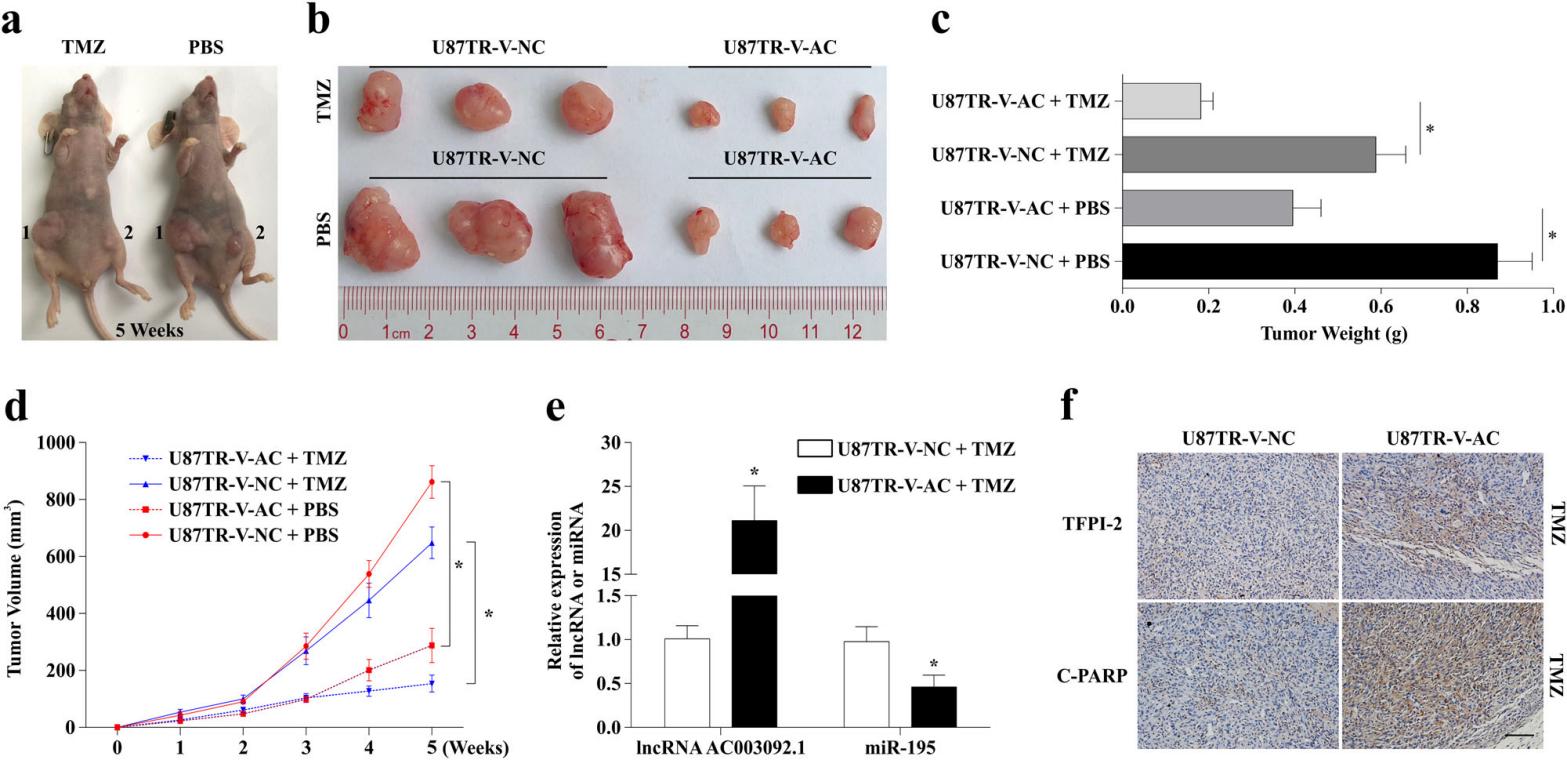
lncRNA AC003092.1 overexpression enhances TMZ sensitivity in vivo. **a, b** Photographs of tumors that developed in xenograft-transplanted nude mice tumor model after injection of U87TR-V-NC (1) or U87TR-V-AC (2) cells at 5 weeks treated with 5 μg/g TMZ or PBS. **c** Weights of tumor xenografts originated from U87TR-V-NC or U87TR-V-AC cells after treatment of TMZ or PBS at 5 weeks. ^*^P < 0.05 compared with U87TR-V-NC with TMZ or PBS treatment. **d** Growth curve of U87TR-V-NC or U87TR-V-AC cells derived subcutaneous tumor xenografts after TMZ or PBS treatment. ^*^P < 0.05 compared with U87TR-V-NC treated with TMZ or PBS. **e** Relative expression of lncRNA AC003092.1 and miR-195 in U87TR-V-NC or U87TR-V-AC cells derived tumor xenograft. **f** Immunohistochemistry analysis of TFPI-2 and C-PARP expression in U87TR-V-NC or U87TR-V-AC cells derived tumor xenograft. Scale bar = 100 μm. Data are presented as mean ± SD of three independent experiments

To further determine the effect of lncRNA AC003092.1 on the sensitivity to TMZ of U87TR cells in vivo, U87TR cells with high expression of lncRNA AC003092.1, U87TR-V-AC, or U87TR-V-NC, by transfecting with V-AC (virus-lncRNA AC003092.1) or V-NC (virus-negative control), were inoculated into the flanks of nude mice. After 6 weeks of inoculation, U87TR-V-AC-injected mice showed a decrease of tumor volume (Fig. 7a–d) and weight (Fig. 7c) compared to U87TR-V-NC mice with PBS, while this trend was more significant with TMZ 5 μg/g treatment (Fig. 7a–d). qRT-PCR analysis of resected tumor tissues suggested that lncRNA AC003092.1 expression was higher in the U87TR-V-AC group than that of the U87TR-V-NC group, while miR-195 expression was significantly downregulated in the U87TR-V-AC group compared with the U87TR-V-NC group with TMZ treatment (Fig. 7e). Moreover, IHC analysis results showed that TFPI-2 and cleaved PARP (C-PARP) expression were increased in the U87TR-V-AC group when compared with the U87TR-V-NC group with TMZ treatment (Fig. 7f).

## Discussion

In this study, we found that lncRNA AC003092.1 and TFPI-2 has low expression in TMZ-resistant U87TR and U251TR cells. Moreover, overexpression of lncRNA AC003092.1 reversed glioblastoma (GB) chemoresistance to TMZ by inhibiting cell proliferation and promoting cell apoptosis with TMZ treatment. Furthermore, we verified that lncRNA AC003092.1 suppressed chemoresistance of GB cells via upregulation of TFPI-2, while miR-195 was involved in lncRNA AC003092.1-mediated upregulation of TFPI-2. Importantly, GB patients with lower lncRNA AC003092.1 expression level had an increased risk of recurrence and worse outcomes, in comparison with patients with higher lncRNA AC003092.1 expression level. These results revealed that lncRNA AC003092.1 has considerable potential in the prognosis and to overcome TMZ resistance treatment of GB patients.

Increasing evidence demonstrates that lncRNA is associated with development of drug resistance and glioblastoma. Studies have shown that knockdown of long noncoding RNA UCA1 suppressed the tamoxifen resistance of breast cancer cells through inhibiting wnt/β-catenin pathway^38^. Knockdown of long noncoding RNA FOXD3 antisense RNA 1 inhibited malignant glioma cells proliferation, migration, and invasion^39^. More recently, Cai et al. have demonstrated that lncRNA MALAT1 could promote chemoresistance through suppressing miR-101 signaling pathway via direct binding in GB cells^40^. In the present study, we screened for TMZ-altered lncRNAs using microarray analysis and found that lncRNA AC003092.1 was significantly decreased in U87TR cells when compared with its parent U87 cells. Similar results were observed in U251TR cells. Additionally, we found that decreased lncRNA AC003092.1 expression level conferred TMZ resistance, while overexpression of lncRNA AC003092.1 enhanced TMZ sensitivity in GB cells. However, the molecular mechanism that leads to the altered differential expression of lncRNA AC003092.1 under TMZ treatment will be further explored in the future.

Subsequently, to determine the cell function affected by lncRNA AC003092.1 in response to TMZ treatment, we conducted EdU, flow cytometry, and TUNEL assay after overexpression of lncRNA AC003092.1. We found that lncRNA AC003092.1 reversed chemoresistance to TMZ by inhibiting cell proliferation and promoting cell apoptosis under TMZ treatment in GB cells. To explore whether lncRNA AC003092.1 exerted its effect on glioblastoma TMZ resistance by its potential target TFPI-2, CNC analysis was conducted. The results showed that lncRNA AC003092.1 had a highly positive correlation with TFPI-2, which was also identified in GB tissue samples. TFPI-2 was considered as a tumor suppressor gene in several types of cancer, including GB^41^. Rao et al. showed that the expression of TFPI-2 inversely correlates during the progression of human gliomas^42^. Similarly, restoration of TFPI-2 in human GB cell line promoted cell apoptosis and inhibited cell proliferation^25,26^. Consistent with these studies, we found that lncRNA AC003092.1 enhanced the sensitivity to TMZ by increasing the expression of TFPI-2 and promoting apoptosis in GB cells.

To date, many studies have shown that lncRNA can function as a miRNA sponge to regulate the expression of miRNA target genes^43–46^. Thus, miRNA may be involved in lncRNA AC003092.1- regulated TFPI-2 expression. The five expressions of miRNA (miR-16-5p, miR-15a-5p, miR-15b-5p, miR-195, and miR-424-5p) with co-prediction combination sequence with lncRNA AC003092.1 and TFPI-2, were measured. Our data showed that miR-195 was significantly downregulated when lncRNA AC003092.1 expression was upregulated. Moreover, luciferase reporter assays and RNA-binding protein immunoprecipitation assays demonstrated that lncRNA AC003092.1 directly interacted with miR-195. Previous studies showed that miR-195 could improve drug sensitivity by enhancing cell apoptosis in hepatocellular carcinoma cells^47^. However, Ujifuku . et al. showed that miR-195 was the most upregulated miRNA in TMZ-resistant cell lines and knockdown of miR-195 displayed a moderate cell-killing effect, and combination with TMZ strongly enhanced the effect in GB cells^48^. Consistent with the above study, we also found that miR-195 was upregulated in U87TR and U251TR cells, concomitant with a decreased level of lncRNA AC003092.1 and TFPI-2. Moreover, the expression of miR-195 was negatively correlated with the expression of lncRNA AC003092.1 and TFPI-2 in clinical patients. Co-transfection of miR-195 and lncRNA AC003092.1 assay, showed that miR-195 reversed the upregulation of TPFI-2 which was induced by overexpression of lncRNA AC003092.1. Luciferase reporter assays also indicated that direct binding of miR-195 to TPFI-2-3’-UTR reduced luciferase activity, while overexpression of lncRNA AC003092.1 attenuates miR-195’s effect and enhanced the luciferase activity. Thus, we concluded that lncRNA AC003092.1 positively regulates posttranscriptional expression of TPFI-2 through miR-195 in GB cells.

In addition, to determine the apoptotic pathway involved in TPFI-2-induced apoptosis with TMZ treatment in GB cells, we built the overexpression of TFPI-2 in U87TR and U251TR cells. Our data showed that TFPI-2 increased the expression of C-Cas 3, C-Cas 8, C-Cas 9, and C-PARP with 50 μg/ml TMZ treatment. This indicates that TFPI-2 sensitizes GB cell lines to TMZ in caspase- dependent apoptosis pathway. The development of multidrug resistance (MDR) by tumor cells is a major obstacle to successful chemotherapy for cancer. One pivotal mechanism by which glioblastoma cells can become resistant to chemotherapy is the increased expression of certain ATP-binding cassette (ABC) transporters which include P-glycoprotein (P-gp, MDR1), multidrug resistance-associated protein (MRP), and breast cancer resistance protein (BCRP)^49–51^. Lu et al. showed that TFPI-2 downregulates multidrug resistance protein in 5-FU-resistant human hepatocellular carcinoma BEL-7402/5-FU cells^27^. Furthermore, studies showed that TFPI-2 dephosphorylates P-glycoprotein/ABCB1 which in turn downregulates its expression and function^52^.

Finally, we identified that a novel lncRNA, lncRNA AC003092.1, enhanced TMZ sensitivity in GB by serving as a ceRNA, through sponging with miR-195 which attenuates the suppression of miR-195 on TFPI-2 and subsequently upregulates TFPI-2 to inhibit cell proliferation and increase apoptosis. This interaction would be considered a potential target for the diagnosis of glioblastoma and outcome assays of TMZ-based therapy. Moreover, miR-195 and TFPI-2 was identified to interact with TMZ resistance in glioblastoma, which explains the poor prognosis of GB patients under TMZ treatment. LncRNA AC003092.1 may exert its effect on GB cells sensitivity to TMZ via other signaling pathways, therefore, further elucidating that the function of lncRNA AC003092.1 is important to overcome TMZ resistance or explain the mechanism via which TMZ resistance develops.

## Materials and methods

### Clinical specimens

All 108 human glioma tissue samples were collected from patients who were undergoing surgery at Zhujiang Hospital (Southern Medical University, China). Glioma specimens, including 75 grade IV (glioblastoma, GB), 5 grade III, 13 grade II, and 15 grade I astrocytoma cases, were classified by two neuropathologists according to the WHO criteria for brain tumors. Samples were immediately frozen in liquid nitrogen after surgical resection. This study was approved by the Ethics Committee of Zhujiang Hospital with informed consents obtained from the patients or their guardians.

### Cell culture and transfection

Human U87 cell line was obtained from the American Type Culture Collection (Manassas, VA, USA) and U251 cell line was purchased from the CLS Cell Lines Service GmbH (Eppelheim, Germany). Their TMZ-resistant lines, U87TR and U251TR, were established and maintained in our laboratory^19,20^. The cells were routinely maintained in Dulbecco’s modified Eagle’s medium (DMEM, Invitrogen, USA) supplemented with 10% fetal bovine serum (FBS, Invitrogen, USA) at 37 ℃ in 5% CO_2_-humidified incubators (Thermo Scientific, Waltham, MA, USA). To maintain the TMZ-resistant phenotype, TMZ (Sigma, San Francisco, CA, USA) dissolved in DMSO with 50μg/ml final concentration was added to the culture medium for U87TR and U251TR cells^19,53^.

For lentiviral stable transfection, cells were plated at 50% confluence in a six-well cell culture cluster with complete growth medium. Eighteen to twenty-four hours post plating, prepare the DNA-medium–polybrene solution, immediately before using as follows: (1) complete growth medium (2 ml for a 60-mm plate) warmed to 37 °C. (2) Plasmid DNA pLVX-IRES-Neo-AC003092.1 (V-AC: virus-lncRNA AC003092.1), pLVX-IRES-Neo-TFPI-2 (V-TFPI-2: virus-TFPI-2), and pLVX-IRES-Neo-vector (V-NC: virus-negative control) multiplicity of infection = 3, gently mix. (3) Polybrene to a final concentration of 5 μg/ml. Gently mix. Remove the medium from the plate and add DNA-medium–polybrene solution to the cells. Incubate the cells at 37 °C for 24 h. Positive transfection cells were selected in 500 μg/ml geneticin for 1 week (G418, Invitrogen, USA). Individual colonies were harvested for the evaluation of gene expression or functional assays.

For siRNA transient transfection, 50–100 nmol/l siRNA-AC003092.1 (Sigma, USA) (Supplementary Table S2), siRNA-TFPI-2 (RiboBio, Guangzhou, China), miR-195 mimics (miR-195), and antagomirs (anti-miR-195) (Genepharma, Shanghai, China) were transfected into GB cells by using Lipofectamine™ RNAiMAX Reagent (Thermo Scientific, Waltham, MA, USA) according to the manufacturer’s protocol. Knockdown efficiency was confirmed by qRT-PCR.

### Co-expression analysis

LncRNA microarray analysis was done between the parental U87 and U87TR GB cell lines^20^. Different expression of long noncoding RNA (lncRNA) and coding RNA (mRNA) was selected for analysis. Construction of the coding–noncoding gene co-expression network was conducted in R environment using RedeR package. Pearson correlation coefficient (PCC) was calculated between a distinct expression of mRNA and lncRNA AC003092.1. Correlations with PCC> = 0.995 were considered as significant. Coding–noncoding networks were created by Cytoscape software^54^.

### RNA isolation, reverse transcription, and quantitative real-time PCR

According to the manufacturer’s instruction, total RNA was isolated from tissue samples or cultured cells using Trizol Reagent (Takara Bio, Shiga, Japan). For lncRNA AC003092.1, first-strand cDNA was generated using the M-MLV Reverse Transcriptase (Promega, Madison, WI, USA). For mRNAs, cDNA was synthesized using the PrimeScript^TM^ RT reagent kit (Takara Bio, Shiga, Japan). To quantitate miRNA (miR-195-5p, miR-15a-5p, miR-15b-5p, miR-424-5p, and miR-16a-5p) expression, total RNA was polyadenylated and reverse transcribed using miRNAs qPCR Quantitation Kit (Gene Copoeia, Guangzhou, China). The RNA expression was measured by qRT-PCR using SYBR Green PCR Master Mix (Takara Bio, Shiga, Japan) which was carried on the ABI 7500 Fast Real Time PCR system (Applied Biosystems, Foster City, CA, USA). The primers used were listed in Supplementary Table S3. U6 snRNA or glyceraldehyde-3-phosphate dehydrogenase (GAPDH) were used as endogenous controls. All data were presented as the means ± SD of at least three independent experiments. Relative expression was determined through relative quantification (2^−ΔΔCt^).

### Protein extraction and western blot analysis

After cells were washed with ice-cold PBS, total proteins were extracted from cells using RIPA buffer supplemented with protease inhibitor cocktail on ice. Cell lysates were centrifuged at 14,000 rpm at 4 °C for 10 min. In total, 40 μg of total protein were separated by 10% SDS-PAGE and electrophoretically transferred to the PVDF membrane (Millipore Corporation, USA). The membranes were blocked with 5% fat-free milk in Tris-buffered saline containing 0.1% Tween-20 (TBST) for 1h at room temperature, followed by incubation with specific antibodies against TFPI-2 (Abcam, USA), caspase-3, cleaved caspase-3, caspase-8, cleaved caspase-8, caspase-9, cleaved caspase-9, and cleaved PARP (Cell Signaling Technology, USA) in 5% fat-free milk in TBST at 4 °C overnight. Membranes were washed and incubated for 2 h with HRP (horseradish peroxidase)-labeled goat-anti-rabbit IgG (Santa Cruz Biotechnology, USA), then detected, and visualized by chemiluminescence. Protein expression was analyzed by the ImageJ software and normalized to that of GAPDH (Cell Signaling Technology, USA).

### TMZ chemosensitivity test and cell viability assay

GB cells after stable transfection or transient transfection were plated in a 96-well plate and treated with different concentrations of TMZ for 48 h. The drug concentrations used were based on earlier studies^19^. After incubating with 10% CCK-8 (Dojindo, Japan) solution fresh medium solution for 2 h, the absorbance at 450 nm was measured using Ultra Multifunctional Microplate Reader (Tecan, Switzerland). IC_50_ values for TMZ were used to evaluate the sensitivities of TMZ in the GB cells. For cell viability assay, GB cells were treated with 50 μg/ml TMZ for 24, 48, 72, 96, and 120 h. Cell growth was determined by the absorbance at 450 nm after 2 h of 10% CCK-8 solution incubation according to the manufacturer’s instructions^6,20^.

### Flow cytometric detection of apoptosis

After transfections, GB cells in six-well plates were treated with TMZ (50 μg/ml) for 48 h, then harvested by 0.25% trypsin, and collected by centrifugation at 1500 rpm for 5 min. To identify apoptotic cells, Annexin V and PI staining was performed with Annexin V-FITC Apoptosis Detection kit (BD Pharmingen, USA) according to the manufacturer’s recommendations. Then cells were analyzed by FACS cytometry (BD Biosciences Inc., Franklin, NJ, USA).

### Fluorescence in situ hybridization (FISH)

LncRNA AC003092.1 FISH probe was synthesized by RiboBio Technology Co. Ltd. (Guangzhou, China). FISH was performed with the kit according to the manufacturer’s protocol (Ribo Bio Tech). Cells were fixed with 4% paraformaldehyde for 10 min at room temperature, and then permeabilized in PBS with 0.5% Triton X-100 on ice for 5 min, followed by pretreatment with pre-hybridization buffer at 37℃ for 30 min. Subsequently, the cells were hybridized with 20 µM using Cy3-labeled RNA of lncRNA AC003092.1 FISH probe mix in a moist chamber at 37℃ overnight. Cells were rinsed thrice in 4× SSC with 0.1% Tween-20 for 5 min at 42 ℃, followed by washing once for 5 min at 42 ℃ in 2 × SSC and then washing once for 5 min at 42 ℃ in 1 × SSC. After hybridization, cells were counterstained with DAPI, and then sections were detected under a fluorescent microscope (Olympus, Tokyo, Japan) and analyzed using Image-Pro Plus 6.0 software (Media Cybernetics, Inc., Rockville, MD, USA).

### Immunohistochemistry staining

Formalin-fixed paraffin-embedded GB or non-tumor samples were sectioned and mounted on microscopic slides. The specimens were incubated with rabbit monoclonal anti-TFPI-2 antibody and anti-C-PARP antibody (1:50 dilutions, Abcam, USA) at 4 °C overnight, followed by 1-h incubation of biotinylated secondary antibody (1:500 dilutions, Santa Cruz Biotechnology, USA) at room temperature. Then the avidin biotinylated peroxidase complex methods were adopted to determine the location and relative expression of target protein to visualize the bound antibodies. Staining intensity was scored as 0 = no staining, 1 = weak staining, 2 = moderate staining, and 3 = strong staining by two independent pathologists manually.

### EdU cell proliferation assay

Cell proliferation was measured by performing 5-ethynyl-2’-deoxyuridine (EdU) incorporation assay with EdU assay kit (Life Technologies Corporation, Carlsbad, CA, USA). Cells were cultured in 24-well plates pre-coated with fresh laminin (Sigma-Aldrich, St. Louis, MO, USA) for adherent culture, and then 10 µM of EdU was added to each well and cells were cultured for an additional 2 h at 37 ℃. Then the cells were fixed with 4% formaldehyde for 30 min at room temperature. After washing, EdU can be detected with a Click-iT Edu kit at room temperature. Subsequently, the cells were stained with Hoechst 33342 for 30 min and visualized using a fluorescent microscope (Olympus, Tokyo, Japan). The EdU-positive cells (green cells) were counted using Image-Pro Plus 6.0 software (Media Cybernetics, Inc., Rockville, MD, USA). The EdU incorporation rate was expressed as the ratio of EdU-positive cells to the total Hoechst 33342 positive cells (blue cells).

### TUNEL cell apoptosis assay

GB cells after transfections were seeded into six-well plates and incubated with TMZ 50 μg/ml or PBS for 48 h. The percentage of TUNEL-positive cells was calculated using a Cell Death Detection Kit (Roche, Mannheim, Germany), according to the manufacturer’s recommendations. Eight randomly selected fields were assessed at 200× magnification with a fluorescent microscope (Olympus, Japan). The images were analyzed with the Image-Pro Plus 6.0 software (Media Cybernetics, Inc., Rockville, MD, USA).

### Dual-luciferase reporter assay

miR-195 response element (wild type or mutated), which contained the lncRNA AC003092.1 or 3′-untranslated regions (3′-UTR) of TFPI-2, was sub-cloned into downstream of the luciferase reporter gene to create pMIR-REPORT-lncRNA AC003092.1-wild-type (Wt) and pMIR-REPORT-3′-UTR-Wt (TFPI-2) plasmid. To test the binding specificity, the corresponding mutant was created with changed region binding site to create pMIR-REPORT-lncRNA AC003092.1-Mut-type (Mut) and pMIR-REPORT-3′-UTR-Mut (TFPI-2) plasmid. The plasmids were co-transfected with miR-control, miR-195, anti-miR-195, anti-miR-control, V-NC, and V-AC (lncRNA AC003092.1) into U87TR cells, and 48 h later, luciferase activity was measured using luciferase assay kit (Promega, Madison, WI, USA). The relative luciferase activity was calculated as firefly fluorescence/Renilla fluorescence.

### RNA-binding protein immunoprecipitation (RIP) assay

RIP assay was conducted by using EZ-Magna RIPTM RNA-binding Protein Immunoprecipitation kit (Millipore Corporation, USA) as per the manufacturer’s recommendations with minor modulation. Antibodies against TFPI-2 (Abcam, USA) and IgG (Millipore Corporation, USA) were used for RIP. The immunoprecipitated RNAs were isolated and detected by reverse transcription PCR and quantitative PCR. Total RNAs (input controls) and IgG were used to demonstrate that the detected signals were the result of RNAs specifically binding to TFPI-2.

### Tumor xenograft model

For this part of the study, 5-week-old male BALB/C nude mice were purchased from the Laboratory Animal Center of Southern Medical University (Guangzhou, China), bred, and maintained in a specific pathogen-free (SPF) facility. For xenograft models, mice were anesthetized with 2.5% isoflurane before tumor implantation. In total, 2×10^6^ U87TR-V-AC and U87TR-V-NC cells were collected and independently injected subcutaneously into the left back and right back of six nude mice, respectively. Once tumors were palpable (50 mm^3^), the tumor-bearing mice were treated with 5 μg/g TMZ in 25% DMSO saline solution by intraperitoneal injection (5 days per week × 3 weeks). Tumor volume were calculated by the following formula: volume = 0.5×(length)×(width)^2,55^. All experimental procedures were conducted in accordance with the National Institutes of Health Guide for the Care and Use of Laboratory Animals and were approved by the Animal Experimental Committee of Southern Medical University.

### Statistical analysis

The results were presented as mean ± standard deviation (SD). Statistical analyses were performed using either one-way analysis of variance (ANOVA) followed by post hoc Tukey’s test or Student’s *t* test. Kaplan–Meier survival curves were generated to evaluate the correlation of lncRNA AC003092.1 expression with survival rate. Mann–Whitney test was used to evaluate the significance of differences between groups in tissue specimens. The relationship between lncRNA AC003092.1, miR-195, and TFPI-2 expression was explored by Pearson correlation. *P* < 0.05 was considered as statistically significant. All statistical analyses were carried out with SPSS 18.0 software (SPSS Inc., Chicago, IL, USA) and GraphPad Prism software 7.0 (GraphPad Software, Inc., San Diego, CA, USA).

## Electronic supplementary material

Supplementary Table S1, Table S2 and Table S3

Supplementary Figure 1

Supplementary Figure 2

Supplementary figure legends
