# Supplementary material for: Long noncoding RNA AC003092.1 promotes temozolomide chemosensitivity through miR-195/TFPI-2 signaling modulation in glioblastoma
Source: Cell Death Dis. 2018 Nov 15;9(12):1139. doi: 10.1038/s41419-018-1183-8 (PMC6237774; doi:10.1038/s41419-018-1183-8)
Supplement: Supplementary file 1 — Supplementary Table S1, Table S2 and Table S3 [file 41419_2018_1183_MOESM1_ESM.docx]

Supplementary Table S1: Predicted miRNAs with binding sites of lncRNA AC003092.1 and TFPI-2 by Starbase 2.0 database (http://starbase.sysu.edu.cn)

| **lncRNA Name** | **miRNA Name** | **mirAccession** | **Target Sites** |
| --- | --- | --- | --- |
| lncRNA AC003092.1 | [hsa-miR-107](http://starbase.sysu.edu.cn/viewMatureMirInfo.php?table=mirLncRNAInteractionsAll&database=hg19&name=hsa-miR-107) | MIMAT0000104 | chr7:93693941-93693963[+] |
| lncRNA AC003092.1 | [**hsa-miR-16-5p**](http://starbase.sysu.edu.cn/viewMatureMirInfo.php?table=mirLncRNAInteractionsAll&database=hg19&name=hsa-miR-16-5p) | MIMAT0000069 | chr7:93693941-93693964[+] |
| lncRNA AC003092.1 | [**hsa-miR-15a-5p**](http://starbase.sysu.edu.cn/viewMatureMirInfo.php?table=mirLncRNAInteractionsAll&database=hg19&name=hsa-miR-15a-5p) | MIMAT0000068 | chr7:93693941-93693964[+] |
| lncRNA AC003092.1 | [**hsa-miR-195-5p**](http://starbase.sysu.edu.cn/viewMatureMirInfo.php?table=mirLncRNAInteractionsAll&database=hg19&name=hsa-miR-195-5p) | MIMAT0000461 | chr7:93693942-93693964[+] |
| lncRNA AC003092.1 | [hsa-miR-497-5p](http://starbase.sysu.edu.cn/viewMatureMirInfo.php?table=mirLncRNAInteractionsAll&database=hg19&name=hsa-miR-497-5p) | MIMAT0002820 | chr7:93693942-93693964[+] |
| lncRNA AC003092.1 | [hsa-miR-103a-3p](http://starbase.sysu.edu.cn/viewMatureMirInfo.php?table=mirLncRNAInteractionsAll&database=hg19&name=hsa-miR-103a-3p) | MIMAT0000101 | chr7:93693941-93693963[+] |
| lncRNA AC003092.1 | [**hsa-miR-15b-5p**](http://starbase.sysu.edu.cn/viewMatureMirInfo.php?table=mirLncRNAInteractionsAll&database=hg19&name=hsa-miR-15b-5p) | MIMAT0000417 | chr7:93693941-93693964[+] |
| lncRNA AC003092.1 | [**hsa-miR-424-5p**](http://starbase.sysu.edu.cn/viewMatureMirInfo.php?table=mirLncRNAInteractionsAll&database=hg19&name=hsa-miR-424-5p) | MIMAT0001341 | chr7:93693943-93693964[+] |

| **miRNA Name** | **Gene Name** | **Position** |
| --- | --- | --- |
| [hsa-miR-26a-5p](http://starbase.sysu.edu.cn/viewMatureMirInfo.php?table=miRNAClipIntersectTargets&database=hg19&name=hsa-miR-26a-5p) | [TFPI2](http://starbase.sysu.edu.cn/viewGeneInfo.php?table=miRNAClipIntersectTargets&database=hg19&name=TFPI2) | chr7:93515770-93515791[-] |
| [**hsa-miR-16-5p**](http://starbase.sysu.edu.cn/viewMatureMirInfo.php?table=miRNAClipIntersectTargets&database=hg19&name=hsa-miR-16-5p) | [TFPI2](http://starbase.sysu.edu.cn/viewGeneInfo.php?table=miRNAClipIntersectTargets&database=hg19&name=TFPI2) | chr7:93515957-93515979[-] |
| [**hsa-miR-15a-5p**](http://starbase.sysu.edu.cn/viewMatureMirInfo.php?table=miRNAClipIntersectTargets&database=hg19&name=hsa-miR-15a-5p) | [TFPI2](http://starbase.sysu.edu.cn/viewGeneInfo.php?table=miRNAClipIntersectTargets&database=hg19&name=TFPI2) | chr7:93515957-93515978[-] |
| [hsa-miR-1297](http://starbase.sysu.edu.cn/viewMatureMirInfo.php?table=miRNAClipIntersectTargets&database=hg19&name=hsa-miR-1297) | [TFPI2](http://starbase.sysu.edu.cn/viewGeneInfo.php?table=miRNAClipIntersectTargets&database=hg19&name=TFPI2) | chr7:93515770-93515786[-] |
| [hsa-miR-17-5p](http://starbase.sysu.edu.cn/viewMatureMirInfo.php?table=miRNAClipIntersectTargets&database=hg19&name=hsa-miR-17-5p) | [TFPI2](http://starbase.sysu.edu.cn/viewGeneInfo.php?table=miRNAClipIntersectTargets&database=hg19&name=TFPI2) | chr7:93515996-93516001[-] |
| [hsa-miR-20a-5p](http://starbase.sysu.edu.cn/viewMatureMirInfo.php?table=miRNAClipIntersectTargets&database=hg19&name=hsa-miR-20a-5p) | [TFPI2](http://starbase.sysu.edu.cn/viewGeneInfo.php?table=miRNAClipIntersectTargets&database=hg19&name=TFPI2) | chr7:93515996-93516001[-] |
| [hsa-miR-433-3p](http://starbase.sysu.edu.cn/viewMatureMirInfo.php?table=miRNAClipIntersectTargets&database=hg19&name=hsa-miR-433-3p) | [TFPI2](http://starbase.sysu.edu.cn/viewGeneInfo.php?table=miRNAClipIntersectTargets&database=hg19&name=TFPI2) | chr7:93515787-93515808[-] |
| [hsa-miR-379-5p](http://starbase.sysu.edu.cn/viewMatureMirInfo.php?table=miRNAClipIntersectTargets&database=hg19&name=hsa-miR-379-5p) | [TFPI2](http://starbase.sysu.edu.cn/viewGeneInfo.php?table=miRNAClipIntersectTargets&database=hg19&name=TFPI2) | chr7:93515938-93515958[-] |
| [hsa-miR-411-5p](http://starbase.sysu.edu.cn/viewMatureMirInfo.php?table=miRNAClipIntersectTargets&database=hg19&name=hsa-miR-411-5p) | [TFPI2](http://starbase.sysu.edu.cn/viewGeneInfo.php?table=miRNAClipIntersectTargets&database=hg19&name=TFPI2) | chr7:93515938-93515960[-] |
| [hsa-miR-382-5p](http://starbase.sysu.edu.cn/viewMatureMirInfo.php?table=miRNAClipIntersectTargets&database=hg19&name=hsa-miR-382-5p) | [TFPI2](http://starbase.sysu.edu.cn/viewGeneInfo.php?table=miRNAClipIntersectTargets&database=hg19&name=TFPI2) | chr7:93515805-93515827[-] |
| [hsa-miR-134-5p](http://starbase.sysu.edu.cn/viewMatureMirInfo.php?table=miRNAClipIntersectTargets&database=hg19&name=hsa-miR-134-5p) | [TFPI2](http://starbase.sysu.edu.cn/viewGeneInfo.php?table=miRNAClipIntersectTargets&database=hg19&name=TFPI2) | chr7:93515998-93516020[-] |
| [hsa-miR-410-3p](http://starbase.sysu.edu.cn/viewMatureMirInfo.php?table=miRNAClipIntersectTargets&database=hg19&name=hsa-miR-410-3p) | [TFPI2](http://starbase.sysu.edu.cn/viewGeneInfo.php?table=miRNAClipIntersectTargets&database=hg19&name=TFPI2) | chr7:93515970-93515990[-] |
| [hsa-miR-138-5p](http://starbase.sysu.edu.cn/viewMatureMirInfo.php?table=miRNAClipIntersectTargets&database=hg19&name=hsa-miR-138-5p) | [TFPI2](http://starbase.sysu.edu.cn/viewGeneInfo.php?table=miRNAClipIntersectTargets&database=hg19&name=TFPI2) | chr7:93516017-93516038[-] |
| [**hsa-miR-195-5p**](http://starbase.sysu.edu.cn/viewMatureMirInfo.php?table=miRNAClipIntersectTargets&database=hg19&name=hsa-miR-195-5p) | [TFPI2](http://starbase.sysu.edu.cn/viewGeneInfo.php?table=miRNAClipIntersectTargets&database=hg19&name=TFPI2) | chr7:93515957-93515976[-] |
| [hsa-miR-497-5p](http://starbase.sysu.edu.cn/viewMatureMirInfo.php?table=miRNAClipIntersectTargets&database=hg19&name=hsa-miR-497-5p) | [TFPI2](http://starbase.sysu.edu.cn/viewGeneInfo.php?table=miRNAClipIntersectTargets&database=hg19&name=TFPI2) | chr7:93515957-93515977[-] |
| [hsa-miR-23a-3p](http://starbase.sysu.edu.cn/viewMatureMirInfo.php?table=miRNAClipIntersectTargets&database=hg19&name=hsa-miR-23a-3p) | [TFPI2](http://starbase.sysu.edu.cn/viewGeneInfo.php?table=miRNAClipIntersectTargets&database=hg19&name=TFPI2) | chr7:93515947-93515952[-] |
| [hsa-miR-519d-3p](http://starbase.sysu.edu.cn/viewMatureMirInfo.php?table=miRNAClipIntersectTargets&database=hg19&name=hsa-miR-519d-3p) | [TFPI2](http://starbase.sysu.edu.cn/viewGeneInfo.php?table=miRNAClipIntersectTargets&database=hg19&name=TFPI2) | chr7:93515995-93516016[-] |
| [hsa-miR-371a-5p](http://starbase.sysu.edu.cn/viewMatureMirInfo.php?table=miRNAClipIntersectTargets&database=hg19&name=hsa-miR-371a-5p) | [TFPI2](http://starbase.sysu.edu.cn/viewGeneInfo.php?table=miRNAClipIntersectTargets&database=hg19&name=TFPI2) | chr7:93515769-93515790[-] |
| [hsa-miR-26b-5p](http://starbase.sysu.edu.cn/viewMatureMirInfo.php?table=miRNAClipIntersectTargets&database=hg19&name=hsa-miR-26b-5p) | [TFPI2](http://starbase.sysu.edu.cn/viewGeneInfo.php?table=miRNAClipIntersectTargets&database=hg19&name=TFPI2) | chr7:93515770-93515790[-] |
| [**hsa-miR-15b-5p**](http://starbase.sysu.edu.cn/viewMatureMirInfo.php?table=miRNAClipIntersectTargets&database=hg19&name=hsa-miR-15b-5p) | [TFPI2](http://starbase.sysu.edu.cn/viewGeneInfo.php?table=miRNAClipIntersectTargets&database=hg19&name=TFPI2) | chr7:93515957-93515978[-] |
| [hsa-miR-340-5p](http://starbase.sysu.edu.cn/viewMatureMirInfo.php?table=miRNAClipIntersectTargets&database=hg19&name=hsa-miR-340-5p) | [TFPI2](http://starbase.sysu.edu.cn/viewGeneInfo.php?table=miRNAClipIntersectTargets&database=hg19&name=TFPI2) | chr7:93515971-93515992[-] |
| [hsa-miR-219a-5p](http://starbase.sysu.edu.cn/viewMatureMirInfo.php?table=miRNAClipIntersectTargets&database=hg19&name=hsa-miR-219a-5p) | [TFPI2](http://starbase.sysu.edu.cn/viewGeneInfo.php?table=miRNAClipIntersectTargets&database=hg19&name=TFPI2) | chr7:93515796-93515816[-] |
| [hsa-miR-590-3p](http://starbase.sysu.edu.cn/viewMatureMirInfo.php?table=miRNAClipIntersectTargets&database=hg19&name=hsa-miR-590-3p) | [TFPI2](http://starbase.sysu.edu.cn/viewGeneInfo.php?table=miRNAClipIntersectTargets&database=hg19&name=TFPI2) | chr7:93515985-93516004[-] |
| [hsa-miR-93-5p](http://starbase.sysu.edu.cn/viewMatureMirInfo.php?table=miRNAClipIntersectTargets&database=hg19&name=hsa-miR-93-5p) | [TFPI2](http://starbase.sysu.edu.cn/viewGeneInfo.php?table=miRNAClipIntersectTargets&database=hg19&name=TFPI2) | chr7:93515995-93516017[-] |
| [hsa-miR-106b-5p](http://starbase.sysu.edu.cn/viewMatureMirInfo.php?table=miRNAClipIntersectTargets&database=hg19&name=hsa-miR-106b-5p) | [TFPI2](http://starbase.sysu.edu.cn/viewGeneInfo.php?table=miRNAClipIntersectTargets&database=hg19&name=TFPI2) | chr7:93515996-93516001[-] |
| [hsa-miR-873-5p](http://starbase.sysu.edu.cn/viewMatureMirInfo.php?table=miRNAClipIntersectTargets&database=hg19&name=hsa-miR-873-5p) | [TFPI2](http://starbase.sysu.edu.cn/viewGeneInfo.php?table=miRNAClipIntersectTargets&database=hg19&name=TFPI2) | chr7:93515837-93515857[-] |
| [hsa-miR-23b-3p](http://starbase.sysu.edu.cn/viewMatureMirInfo.php?table=miRNAClipIntersectTargets&database=hg19&name=hsa-miR-23b-3p) | [TFPI2](http://starbase.sysu.edu.cn/viewGeneInfo.php?table=miRNAClipIntersectTargets&database=hg19&name=TFPI2) | chr7:93515947-93515952[-] |
| [hsa-miR-23c](http://starbase.sysu.edu.cn/viewMatureMirInfo.php?table=miRNAClipIntersectTargets&database=hg19&name=hsa-miR-23c) | [TFPI2](http://starbase.sysu.edu.cn/viewGeneInfo.php?table=miRNAClipIntersectTargets&database=hg19&name=TFPI2) | chr7:93515947-93515952[-] |
| [hsa-miR-223-3p](http://starbase.sysu.edu.cn/viewMatureMirInfo.php?table=miRNAClipIntersectTargets&database=hg19&name=hsa-miR-223-3p) | [TFPI2](http://starbase.sysu.edu.cn/viewGeneInfo.php?table=miRNAClipIntersectTargets&database=hg19&name=TFPI2) | chr7:93515812-93515835[-] |
| [hsa-miR-374b-5p](http://starbase.sysu.edu.cn/viewMatureMirInfo.php?table=miRNAClipIntersectTargets&database=hg19&name=hsa-miR-374b-5p) | [TFPI2](http://starbase.sysu.edu.cn/viewGeneInfo.php?table=miRNAClipIntersectTargets&database=hg19&name=TFPI2) | chr7:93516006-93516028[-] |
| [hsa-miR-374a-5p](http://starbase.sysu.edu.cn/viewMatureMirInfo.php?table=miRNAClipIntersectTargets&database=hg19&name=hsa-miR-374a-5p) | [TFPI2](http://starbase.sysu.edu.cn/viewGeneInfo.php?table=miRNAClipIntersectTargets&database=hg19&name=TFPI2) | chr7:93516006-93516028[-] |
| [hsa-miR-20b-5p](http://starbase.sysu.edu.cn/viewMatureMirInfo.php?table=miRNAClipIntersectTargets&database=hg19&name=hsa-miR-20b-5p) | [TFPI2](http://starbase.sysu.edu.cn/viewGeneInfo.php?table=miRNAClipIntersectTargets&database=hg19&name=TFPI2) | chr7:93515995-93516017[-] |
| [hsa-miR-106a-5p](http://starbase.sysu.edu.cn/viewMatureMirInfo.php?table=miRNAClipIntersectTargets&database=hg19&name=hsa-miR-106a-5p) | [TFPI2](http://starbase.sysu.edu.cn/viewGeneInfo.php?table=miRNAClipIntersectTargets&database=hg19&name=TFPI2) | chr7:93515996-93516001[-] |
| [hsa-miR-503-5p](http://starbase.sysu.edu.cn/viewMatureMirInfo.php?table=miRNAClipIntersectTargets&database=hg19&name=hsa-miR-503-5p) | [TFPI2](http://starbase.sysu.edu.cn/viewGeneInfo.php?table=miRNAClipIntersectTargets&database=hg19&name=TFPI2) | chr7:93515957-93515979[-] |
| [**hsa-miR-424-5p**](http://starbase.sysu.edu.cn/viewMatureMirInfo.php?table=miRNAClipIntersectTargets&database=hg19&name=hsa-miR-424-5p) | [TFPI2](http://starbase.sysu.edu.cn/viewGeneInfo.php?table=miRNAClipIntersectTargets&database=hg19&name=TFPI2) | chr7:93515957-93515978[-] |
| [hsa-miR-224-5p](http://starbase.sysu.edu.cn/viewMatureMirInfo.php?table=miRNAClipIntersectTargets&database=hg19&name=hsa-miR-224-5p) | [TFPI2](http://starbase.sysu.edu.cn/viewGeneInfo.php?table=miRNAClipIntersectTargets&database=hg19&name=TFPI2) | chr7:93515996-93516016[-] |

Supplementary Table S2: Sequence of siRNAs and control used for knockdown of lncRNA AC003092.1 in GB cells

| **siRNA** | **Sequence (5’-3’)** | |
| --- | --- | --- |
| LncRNA AC003092.1-siRNA#1 | Sense: GUAAUCCAGCGAAUCUGGA | Antisense: UCCAGAUUCGCUGGAUUAC |
| LncRNA AC003092.1-siRNA#2 | Sense:  CAGCAAUCAACAUAAUCAA | Antisense:  UUGAUUAUGUUGAUUGCUG |
| LncRNA AC003092.1-siRNA#3 | Sense:  CAGAACAAGAACCUAAGAU | Antisense:  AUCUUAGGUUCUUGUUCUG |
| Control-siRNA | Sense:  UUCUCCGAACGUGUCACGUTT | Antisense:  ACGUGACACGUUCGGAGAATT |

Supplementary Table S3: The primers used in qRT-PCR analysis in GB cells

| **LncRNA/mRNA** | **Forward primer** | **Reverse primer** |
| --- | --- | --- |
| LncRNA AC003092.1 | TTAGCAGCAAACCCAGAAC | TGCTGAGGATACATGACGAA |
| U6 | CTCGCTTCGGCAGCACA | AACGCTTCACGAATTTGCGT |
| TFPI2 | GCCTGAGAACTTTGAATGATGCTG | GGCCCTGTGTTTCTTATGTATCCTG |
| GAPDH | GAGGTGATAGCATTGCTTTCG | CAAGTCAGTGTACAGGTAAGC |
