# Supplementary figures and images for: Long noncoding RNA AC003092.1 promotes temozolomide chemosensitivity through miR-195/TFPI-2 signaling modulation in glioblastoma
Source: Cell Death Dis. 2018 Nov 15;9(12):1139. doi: 10.1038/s41419-018-1183-8 (PMC6237774; doi:10.1038/s41419-018-1183-8)

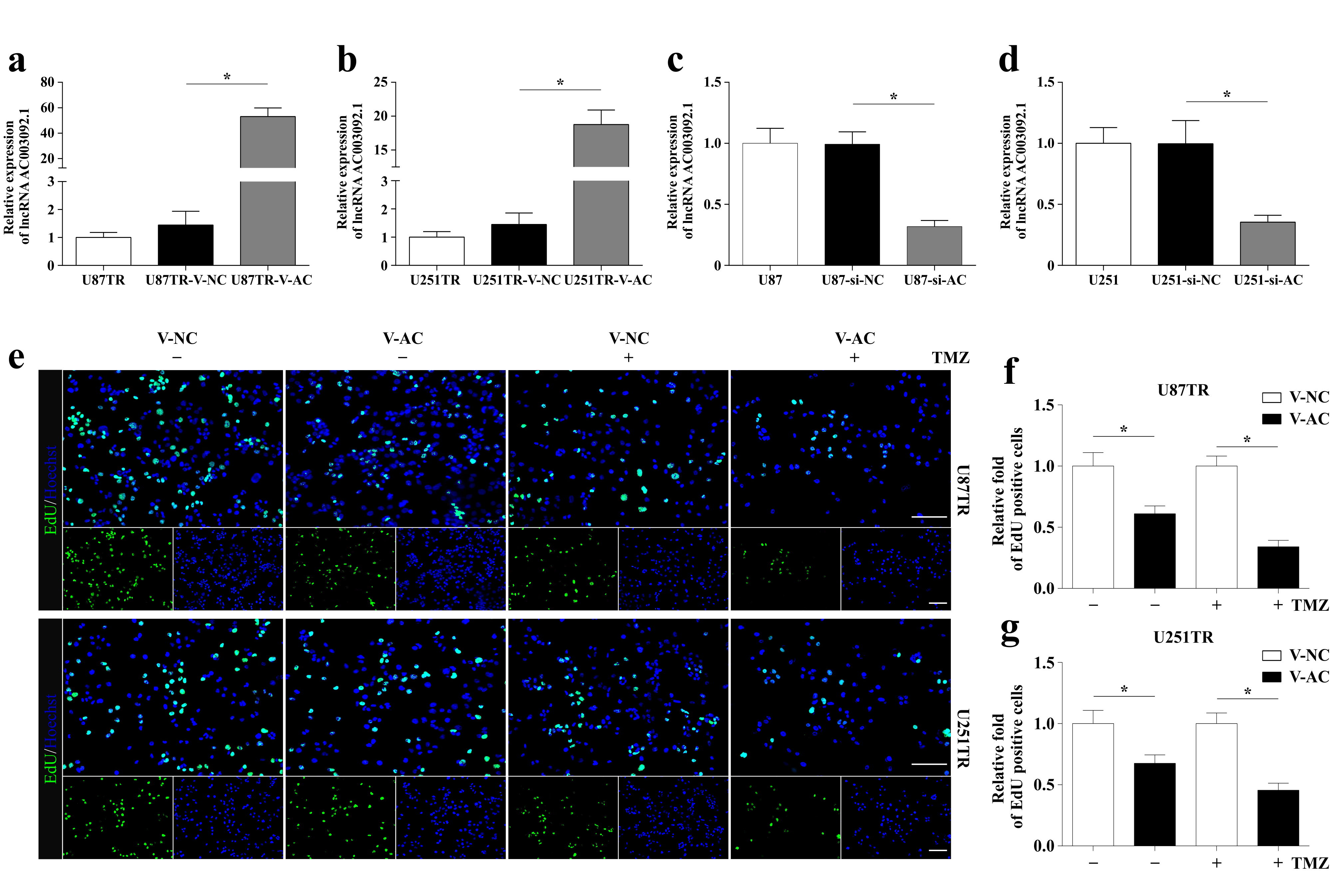

Supplement: Supplementary file 2 — Supplementary Figure 1 [file 41419_2018_1183_MOESM2_ESM.tif]

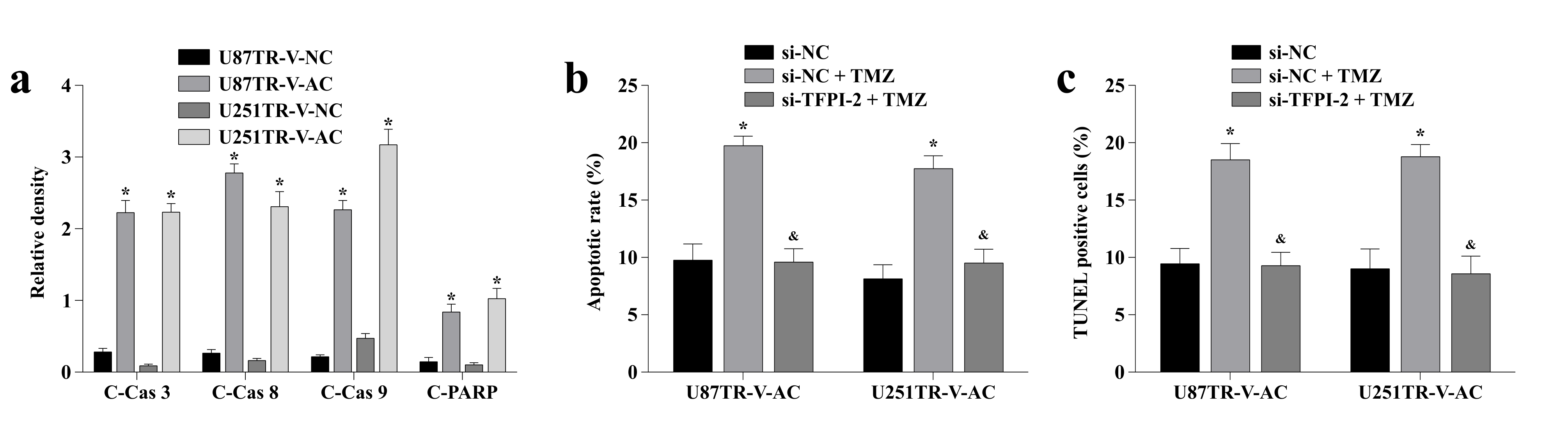

Supplement: Supplementary file 3 — Supplementary Figure 2 [file 41419_2018_1183_MOESM3_ESM.tif]
