# Supplementary material for: Long noncoding RNA AC003092.1 promotes temozolomide chemosensitivity through miR-195/TFPI-2 signaling modulation in glioblastoma
Source: Cell Death Dis. 2018 Nov 15;9(12):1139. doi: 10.1038/s41419-018-1183-8 (PMC6237774; doi:10.1038/s41419-018-1183-8)
Supplement: Supplementary file 4 — Supplementary figure legends [file 41419_2018_1183_MOESM4_ESM.docx]

**Supplementary figure legends**

Supplementary Figure 1 lncRNA AC003092.1 expression in U87TR (**a**) and U251TR (**b**) cells after lentiviral stable transfection with V-AC or V-NC measured by qRT-PCR. ^*^*P*<0.05 compared with V-NC group cells. lncRNA AC003092.1 expression in U87 (**c**) and U251(**d**) cells after siRNA-lncRNA AC003092.1 (si-AC) or si-RNA control (si-NC) transfection determined by qRT-PCR. ^*^*P*<0.05 compared with si-NC group cells. (**e-g**) Effect of lncRNA AC003092.1 expression on cell proliferation with or without TMZ treatment determined by EdU assay. (**e**) Representative pictures of EdU staining. The percentage of EdU positive cells in U87TR (**f**) and U251TR (**g**) V-AC or V-NC groups with or without TMZ treatment. ^*^*P*<0.05 compared with V-NC group cells with or without TMZ treatment.

Supplementary Figure 2 Statistical analysis of western blot in U87TR and U251TR cells after V-NC or V-AC treated (**a**). ^*^*P*<0.05 compared with V-NC group cells. Statistical analysis of flow cytometric (**b**) and TUNEL assays (**c**) after si-TFPI-2 or si-NC and V-AC co-transfection in U87TR and U251TR cells with or without TMZ treatment. ^*^*P*<0.05 compared with si-NC group cells; ^&^*P*<0.05 compared with si-NC group cells with TMZ treatment.
